# Learning Humanities and Ethics of Aging Through the Lens of an Avatar Creation

**DOI:** 10.1093/geroni/igab046.2217

**Published:** 2021-12-17

**Authors:** Gay Hanna, Yoon Chung Kim

**Affiliations:** Georgetown University, Washington DC, District of Columbia, United States

## Abstract

The main goal of teaching the humanities and ethics of aging is to understand the perspectives of older individuals as they address the challenges and opportunities presented across the aging spectrum. To encourage understanding of this humanistic and ethical process, students were given an assignment to select a profile of an older person with pre-selected characteristics that they then develop into their avatar, a virtual companion, to accompany them through the course. This assignment included three iterations of the avatar narrative related to what is studied in class around major life transition points related to work, housing, and end of life. These assignments included the creation of Mind Maps which illustrate their avatar’s ongoing concerns related to their environment including their social determinants of health. The avatar’s formative development throughout the course brought forward discussions around identity, safety, autonomy, and person-centeredness in terms of gerontological practice and policy.

